# The Influence of a Nanoemulsion of Cardamom Essential Oil on the Growth Performance, Feed Utilization, Carcass Characteristics, and Health Status of Growing Rabbits under a High Ambient Temperature

**DOI:** 10.3390/ani13182990

**Published:** 2023-09-21

**Authors:** Rehab F. S. A. Ismail, Mahmoud A. E. Hassan, Mahmoud Moustafa, Mohammed Al-Shehri, Reem S. Alazragi, Hanan Khojah, Ali Ali El-Raghi, Sameh A. Abdelnour, Alaa M. A. Gad

**Affiliations:** 1 Department of Animal Production, Faculty of Agriculture, Mansoura University, Mansoura 35516, Egypt; 2 Animal Production Research Institute (APRI), Agriculture Research Center, Ministry of Agriculture, Dokki, Giza 12619, Egypt; m.hassan55213@gmail.com; 3 Department of Biology, Faculty of Science, King Khalid University, Abha 62217, Saudi Arabia; 4 Department of Biochemistry, College of Science, University of Jeddah, Jeddah 23445, Saudi Arabia; rsalazragi@uj.edu.sa; 5 Department of Pharmacognosy, College of Pharmacy, Jouf University, Sakaka 72388, Saudi Arabia; 6 Department of Animal, Poultry and Fish Production, Faculty of Agriculture, Damietta University, Damietta 34517, Egypt; 7 Department of Animal Production, Faculty of Agriculture, Zagazig University, Zagazig 44519, Egypt

**Keywords:** essential oil, nanoemulsion, rabbit, heat stress, growth, inflammatory responses, immunity

## Abstract

**Simple Summary:**

Heat stress is one of the most severe consequences of climate change, affecting the rabbit production sector. The exposure of growing rabbits to heat stress compromises animal health and welfare, suppresses immunity, and increases morbidity. Dietary interventions by applying nanotechnology could effectively mitigate these negative effects of heat stress in growing rabbits. In this study, we tested various levels of a nanoemulsion of cardamom essential oil (NCEO) and its role in mitigating heat stress in growing V-line rabbits via promoting health, immunity, and growth. Added NCEO (300 mg/kg) improved the growth indices, feed efficiency, redox balance, and immunity and reduced the growing rabbits’ inflammatory responses during the summer.

**Abstract:**

Recently, nanotechnology approaches have been employed to enhance the solubility, availability, and efficacy of phytochemicals, overcoming some industrial obstacles and natural biological barriers. In this regard, 120 clinically healthy growing V-line rabbits (5 weeks old) reared during the summer season were divided randomly into four equal experimental groups (30 rabbits each). The first group received a basal diet without the supplementation of the nanoemulsion of cardamom essential oil (NCEO) (0 g/kg diet) and served as a control (NCEO 0). The other groups were given diets containing NCEO at levels of 150 (NCEO 150), 300 (NCEO 300), and 600 (NCEO 600) mg/kg diet, respectively. The growth performance (higher LBW and ADG), feed utilization (lower FCR), dressing percentage, and relative weight of the liver were improved significantly in the NCEO-treated groups compared to the control group. Moreover, the dietary treatment significantly decreased the rectum temperature and respiration rate, minimizing the 350 and 325 mg NECO/kg diets. The erythrocyte count, hematocrit, and hemoglobin concentration were significantly increased (*p* < 0.05), while white blood cells were significantly diminished (*p* = 0.0200) in the NCEO300 and NCEO600 groups compared to the control group. Treatment with 300 or 600 mg NCEO/kg significantly increased the blood serum total protein and albumin compared to the control group. Meanwhile, the liver enzymes (AST and ALT), uric acid, and creatinine concentrations decreased significantly in the NCEO300 group compared to the control group. The concentrations of triglycerides and total cholesterol were reduced significantly by the dietary treatment. The total antioxidant capacity, dismutase activity, and glutathione concentration were significantly higher, while the malondialdehyde and protein carbonyl levels were significantly lower in the NCEO300 group than in the control. The inflammatory responses and immunity statuses were improved in the blood serum of the NCEO-treated rabbits compared to the control. Heat-stress-induced pathological perturbations in renal/hepatic tissues and NCEO co-treatment successfully re-established and recovered near-control renal–hepatic morphology. In conclusion, a dietary supplementation of NCEO (300 mg/kg) could effectively enhance growing rabbits’ growth indices, feed efficiency, redox balance, immunity, and inflammatory responses during the summer.

## 1. Introduction

Heat stress (HS) is a condition where rabbits cannot balance their heat production and emissions [1]. During the summer in Egypt, the elevated ambient temperature can easily induce HS in rabbits, which causes a series of unfavorable impacts on rabbit productivity [2]. As is known, HS has multiple adverse impacts on rabbits’ health statuses and their production performance. It has been revealed that HS is responsible for a 9–12% increase in mortality rate, 20–25% reductions in average daily weight gain, and 8–15% decreases in feed conversion ratio, as well as negatively affecting the carcass traits and quality of meat in growing rabbits [3,4]. Thus, HS poses a significant challenge for the rabbit industry, made worse by recent global warming [1]. Several previous studies have investigated prospective mitigation strategies, and nutritional manipulation has proven to be a dynamic mitigation approach [4,5]. The dietary supplementation of animal diets with herbs or spices and their derivatives has provided appropriate evidence as a potential alternative to antibiotics for alleviating the negative effects of HS during summer. These products have gained acceptability among consumers as natural feed additives [6,7,8]. Previous studies have revealed significant effects of adding medical herbs and spices (phytogenic additives) to growing rabbits’ diets on growth indices and general health status [9,10]. Cardamom, as a common spice, is widely used for flavoring and culinary purposes worldwide. Cardamom consists of seeds of the dried fruits of Elettaria cardamomum. Moreover, cardamom fruit contains lipids, phytosterols, sterols, phenolic acids, and essential oils. Cardamom essential oil is rich in limonene, α-terpinyl acetate, α-pinene, myrcene, α-terpineol, sabinene, terpinolene, linalyl acetate, linalool, and phellandrene [11]. Generally, multiple published reports have clarified that the cardamom essential oil (CEO) isolated from fruits has antispasmodic, anti-inflammatory, and antimicrobial activities [12,13]. The anti-inflammatory and antioxidant effects of cardamom essential oil have been confirmed in several investigations [14,15]. Despite these beneficial effects of cardamom essential oil, its low solubility, permeability, storage instability, and bioavailability have limited its use in some pharmaceutical uses. Nanoemulsions are a nanotechnology method for improving the previous features of essential oils. In this sense, recent studies have confirmed that converting phytochemicals into nano-form can endow them with several biological activities, making them more convenient for dealings under industrial conditions and more mobile in biological systems [16,17]. Based on the anti-inflammatory, anti-microbial, and antioxidant properties of cardamom essential oil, we theorized that the nano-form of cardamom essential oil (NCEO) could potentially reduce the adverse effects of HS in growing rabbits. Therefore, the current study aims to examine the effect of dietary NCEO supplementation on the growth indices, carcass traits, physiological responses, hemato-biochemical variables, redox balance, inflammatory responses, and immunity statuses of V-line growing rabbits exposed to a high ambient temperature.

## 2. Materials and Methods

The current study was conducted at the Rabbit Research Unit, Faculty of Agriculture, Zagazig University, Egypt, from July to August. All the procedures and protocols used in this study were permitted and approved by the scientific committee of the IACUC of Zagazig University (Approval number: ZU-IACUC/2/F/174/2022).

### 2.1. Preparation of NCEO

The cardamom essential oil (CEO) was purchased from the Pure Life Company in Giza, Egypt. A single layer of NCEO, oil-in-water, was prepared [18]. In brief, CEO prepared nano-emulsions by slowly dropping water (approximately 10 mL) with a surfactant (tween 80) appointed by a magnetic stirrer at 25 °C. The water-dropping rate was preserved at 1.0 mL/min. After that, the nano-emulsion was scattered through an ultrasonic bath for 30 min. Additionally, it was homogenized by employing an ultrasonic probe (integrated with a homogenizer (Sonics Vibra-cell™, Model VC 505, Inc., Newtown, CT, USA)) under the ulterior conditions: timer: 5 min, amplitude: 60%, and pulser: 1 s ON/1 s OFF to yield NCEO.

### 2.2. Physicochemical Properties of NCEO

A transmission electronic microscope (TEM, JEOL JEM-2100, Tokyo, Japan) was used to visualize the internal morphology of the freshly prepared NCEO at 200 kV. The completed image capture analysis process was run through the Digital Micrograph and Soft Imaging Viewer software (Gatan Microscopy Suite Software, version 2.11.1404.0). The average vesicular sizes (Z-average) of the nano-emulsion, the surface charge of the nanoemulsion particles (Z-potential), and the polydispersity index (PDI) were measured using a Zetasizer Nano ZS analyzer.

### 2.3. Animals, Experimental Design and Diets

A total of 120 male V-line growing weaned rabbits (five weeks of age) with an average body weight of 653.91 ± 1.92 g were divided randomly into four homogenous groups (30 rabbits/group) for eight consecutive weeks during the summer season (July and August). The experimental groups were as follows: the first group was given a basal diet without the supplementation of the cardamom essential oil nanoemulsion (NCEO) (0 g/kg diet) and served as a control (NCEO 0). The other three treated groups received basal diets supplemented with 150, 300, and 600 mg of an NCEO/kg diet, NCEO150, NCEO300, and NCEO600, respectively. The NCEO was incorporated into the diet during the process of manufacturing. After preparing and characterizing the NCEO at the Electron Microscopy Unit, Mansoura University, the samples were transferred and stored at 4 °C, pending supplementation. When the rabbit diets were prepared, the NCEO was mixed with the pellet diets in a commercial mixer at various levels according to the study procedure. The animals were kept in galvanized wire battery cages (50 cm length × 50 cm width × 40 cm high). All the rabbits were kept in a naturally ventilated building under similar management and hygiene conditions. A pelleted diet and freshwater were offered ad libitum during the experimental period. According to Blas and Wiseman, the basal diet (Table 1) was free of antibiotics and incorporated to cover the nutritional requirements [19].

### 2.4. Meteorological Parameters

The ambient environmental temperatures (°C) and relative humidity were assessed daily during the experimental period via an automatic Thermo hygrometer (Dostmann GmbH and Co. KG, Wertheim, Germany) set in the rabbitry. The temperature–humidity index (THI) was measured using the aforementioned data according to Marai et al. [20]:

$$THI=dp-\left[\left(0.31-0.31(\frac{RH}{100})\right)\times (dp-14.4)\right]$$

where dp is the dry bulb temperature in Celsius and RH is the relative humidity. The THI values were classified as follows: <27.8 (absence of HS); 27.8 to 28.9 (moderate HS); 29.0 to 30.0 (severe HS); and >30.0 (very severe HS).

### 2.5. Growth Performance and Physiological Responses

During the experimental period, each rabbit’s live body weight and feed intake were measured biweekly. Then, the average daily weight gain and feed conversion ratio (FCR; g feed/g gain) were estimated based on the collected data by period. The rectal temperature in Celsius (RT; °C) and respiratory rate (RR; breath/min) were measured biweekly. The RT was assessed by inserting a digital thermometer to a depth of 4 cm into the rectum. Meanwhile, using a stopwatch, the RR was estimated by optically calculating the number of motions of the animal’s flanks resting for 1 min.

### 2.6. The Carcass Traits

At the end of the experimental period, ten rabbits were selected randomly from each group, fasted for 12 h, and slaughtered immediately after weighing according to the Islamic method. After complete bleeding, the pelt, tail, and viscera were isolated as non-edible parts. Thereafter, the carcass and edible parts (heart, kidney, and liver), as well as the head, spleen, and lung, were weighed as a percentage of the live body weight. The dressing percentage was the ratio of the hot-dressed carcass weight to the weight of the live animal (pre-slaughter weight), expressed as a percentage [21]. In addition, the cecum length (cm) was registered.

### 2.7. Blood Hematology

Ten blood samples were collected from the slaughtered rabbits at the termination of the experimental period, placed in sterile tubes, and divided into two subsamples. The first one was used to evaluate the hematological attributes according to Schalm et al. [22] via an automated hematology analyzer (Hospitex Hema Screen 18, Sesto Fiorentino, Italy), while the second subsample was left to clot and centrifuged for 15 min at 1507× *g*, then the serum samples were separated and stocked at −20 °C until examination.

### 2.8. Blood Constituents

Blood serum metabolites, including the total protein (TP), albumin, liver enzymes (aspartate aminotransferase, AST; alanine transaminase, ALT; and gamma-glutamyl transferase, GGT), total bilirubin, triglyceride, total cholesterol, low-density lipoprotein, and high-density lipoprotein were estimated spectrophotometrically using commercial kits provided from the Bio-diagnostic Company (Giza, Egypt), following the manufacturer’s instructions. The very low-density lipoprotein (VLDL) values were estimated according to the previous technique of [23], based on the total triglycerides. The difference between the total protein and albumin was used for determining the serum globulin.

For assessing the oxidative stress and antioxidant parameters, the activities of superoxide dismutase (SOD) and the total antioxidant capacity (TAC) [8], as well as the levels of glutathione concentration (GSH) [24], malondialdehyde (MDA) [25], and protein carbonyl (PC) [26], in the rabbit serum were measured using commercial kits and a spectrophotometer (Shimadzu, Kyoto, Japan). Immunoglobulins (IgG and IgM) were assayed according to the previous methodology described in the report of Loizou et al. [27]. Concerning the inflammatory responses, including interferon-gamma (IFN-γ) and interleukin-4 (IL-4), these were detected in the rabbit serum following the protocol of Houssen et al. [28]. The serum nitric oxide concentrations were determined using the Griess reagent [29]. The interferon-γ and interleukin-4 levels were quantitated using an Elisa kit assay following the producer’s instructions (MyBioSource, San Diego, CA, USA).

### 2.9. Histological Investigation

At the end of the experimental period, the liver and kidney histology of the slaughtered rabbits were studied according to the previous method [30]. In brief, the tissue samples were fixed in buffered formalin saline (10%). The tissue samples were dehydrated using ethyl alcohol at increasing concentrations (from 70% to absolute), then embedded in paraffin after infiltration with xylene. The paraffin sections were cut using a microtome. Finally, they were stained by haematoxylin and eosin staining for light microscopy (Olympus CX31).

### 2.10. Statistical Analysis

The data were edited in Microsoft Excel version16 (Microsoft Corporation, Redmond, WA, USA). A Shapiro–Wilk test was conducted to check for normality, as described by Razali and Wah [31]. A MIXED procedure (PROC MIXED; SAS Institute, 2012) was used for assessing the growth performances, feed intake, feed conversion ratio, carcass indices, hemato-biochemical parameters, redox status, immunity parameters, and inflammatory responses. The statistical model introduced individual rabbits as a random factor, while NCEO levels were introduced as a fixed factor. Multiple comparisons between means were conducted according to Duncan’s multiple range tests [32]. Statistical significance was accepted when the *p*-value was less than 0.05.

## 3. Results

### 3.1. Meteorological Parameters

The results of the meteorological parameter results during the experimental period are presented in Table 2. The overall averages of the ambient temperature (AT) and relative humidity (RH), as well as the calculated temperature–humidity index (THI), were 31.16 ± 0.07 °C, 72.13 ± 0.21%, and 29.70 ± 0.04, respectively. The values of the THI indicated that the newly weaned rabbits suffered from severe heat stress.

### 3.2. Characterization of NECO Formation

**Figure 1:**
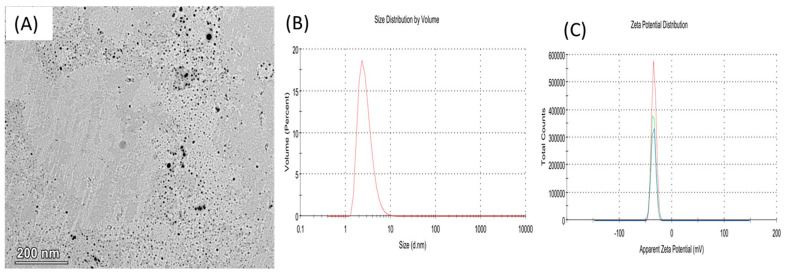
The characterization of NECO by TEM (**A**) shows a nearly spherical morphology. At the same time, the value of zeta size was 4.454 nm (**B**) and the value of zeta potential distribution was −35.1 mV (**C**).

Figure 1A shows the TEM image for NCEO. The image shows little or no aggregation and a nearly spherical particle morphology of the NECO observed. The values of the zeta size and zeta potential of NECO were found to be 4.454 nm and −35.1 mV, respectively (Figure 1B,C).

### 3.3. Growth Performance and Feed Utilization

The effects of the dietary supplementation of NCEO on the growth indices and feed efficiency of the growing rabbits during the experimental period are presented in Table 3. Generally, the values of the live body weight and daily weight gain were improved significantly in all the treated groups compared to the control (*p* < 0.05), maximizing in the NECO600 group. Concerning feed utilization, the dietary treatment had no significant effects on feed intake (*p* = 0.7077). Meanwhile, the NCEO300 and NCEO600 groups were superior to the control in terms of feed conversion ratio (*p* < 0.05), minimizing in the NCEO600-treated group.

### 3.4. Physiological Responses

**Figure 2:**
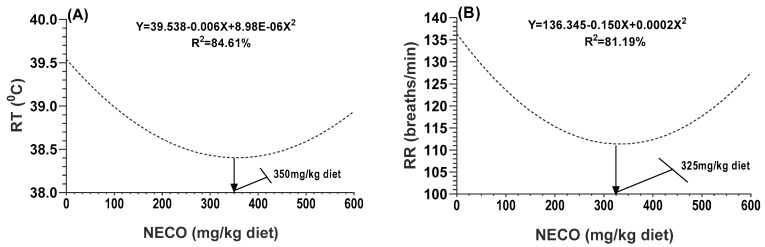
A polynomial regression analysis between dietary levels of NECO and rectal temperature ((**A**) RT; °C), and respiration rate. ((**B**) RR; breaths/min). Rabbit-fed diets have different levels of nanoemulsion of cardamom essential oil at 0 (NCEO0), 150 (NCEO150), 300 (NCEO300), and 600 (NCEO600) mg/kg diet, respectively.

The results of the physiological responses, including the rectal temperature (RT) and respiration rate (RR), are illustrated in Figure 2A,B, respectively. The polynomial regression analysis indicated that the dietary treatment quadratically reduced the RT and RR. The lowest values were coupled by doses of 350 and 325 mg of the NECO/kg diet for the RT and RR, respectively.

### 3.5. Carcass Traits

The results of the carcass characteristics are illustrated in Table 4. The dietary administration of NCEO significantly affected the dressing percentage and relative weights of the liver and edible giblets (*p* = 0.090, 0.0239, and 0.0104, respectively). The dressing percentage was significantly higher in the NECO300 group compared to the control (*p* < 0.05). Meanwhile, the liver and edible giblets’ relative weight increased significantly in the NECO300 and NECO600 groups compared to the control group (*p* < 0.05). The relative weights of the kidney, heart, spleen, lung, and cecum length did not differ significantly between all the treated groups and the control (*p* > 0.05).

### 3.6. Blood Hematology

The effects of the NCEO supplementation on the hematological parameters are illustrated in Table 5. The values of red blood corpuscles, hemoglobin, and hematocrit were significantly higher in the NCEO600 group than in the control group (*p* < 0.05). The platelet count was significantly higher in the NCEO150 and NCEO300 groups than in the control group (*p* < 0.05). The content of white blood cells in the blood was decreased significantly by the dietary treatment (*p* = 0.0200), minimizing in the NCEO300 and NCEO600 groups (*p* < 0.05). No significant differences (*p* > 0.05) were shown in the MCV, MCH, MCHC, and leucocyte fractions between all the treated groups and the control.

### 3.7. Serum Biochemistry

As presented in Table 6, most blood metabolites were statistically (*p* < 0.05 or 0.001) affected by the dietary treatment. The serum total protein and globulin concentration increased significantly in the NCEO300 and NCEO600 groups compared to the control (*p* < 0.05). In contrast, the albumin concentration was significantly higher in the NCEO300 group than in the control and NCEO150 groups (*p* < 0.05). Concerning liver functions, the activities of ALT and AST were significantly diminished (*p* < 0.05) in the blood serum of the rabbits who received high doses of NCEO (300 or 600 mg/kg diet) compared to the control group. Non-significant differences were observed between the treated groups and the control for the total bilirubin and glutamyl transferase (*p* > 0.05). In terms of kidney function, the uric acid and creatinine concentrations were significantly lower in the treated groups than in the control (*p* < 0.05). Concerning blood serum glucose, it was decreased significantly by the dietary treatment (*p* = 0.0001), being significantly lower in the NCEO300 or NCEO600 group than the control group (*p* < 0.05). Regarding the blood lipid profile, the triglycerides, total cholesterol, and low-density lipoprotein values decreased significantly (*p* = 0.0005, 0.0001, and 0.0197, respectively) in response to the dietary treatment. In contrast, the high-density lipoprotein was significantly higher in the treated groups than the control group (*p* < 0.05).

### 3.8. Redox Balance

The results in Table 7 indicate that there were significant positive effects of the NCEO essential oil on the redox status concerning the concentrations of the total antioxidant capacity (TAC), superoxide dismutase (SOD), glutathione (GSH), malondialdehyde (MDA), and protein carbonyl (PC). The concentrations of TAC and GSH were significantly higher in the NCEO600 group than in the control and other treated groups (*p* < 0.05). Meanwhile, the SOD activity increased significantly in the NCEO300 group (*p* < 0.05) compared to the control and other NCEO-treated groups. Concerning oxidative stress, lipid and protein peroxidation were depressed significantly due to the dietary supplementation of NCEO. The MDA and PC concentrations were significantly lower in the NCEO300 groups than in the control (*p* < 0.05).

### 3.9. Inflammatory Responses and Immunity Status

The effects of the dietary administration of NCEO on the inflammatory responses, concerning the interferon γ (INF-γ), interleukin-4 (IL-4), and nitric oxide (NO) of the growing rabbits, are shown in Table 8. The dietary treatment significantly affected all the inflammatory cytokines (*p* = 0.0164, 0.0032, and 0.0012, respectively). Compared to the control group, both INF-γ and IL-4 decreased significantly in the NCEO300 and NCEO600 groups (*p* < 0.05). Meanwhile, the NO concentrations were significantly higher in all the treated groups compared to the control group (*p* < 0.05). Regarding the immunity status, the present results showed significant effects of NCEO on immunological patterns, such as immunoglobulin M (IgM) and immunoglobulin G (IgG), which were significantly (*p* < 0.05) improved in the blood serum of the rabbits that received NCEO compared to their counterparts in the control group.

### 3.10. Histological Investigation

**Figure 3:**
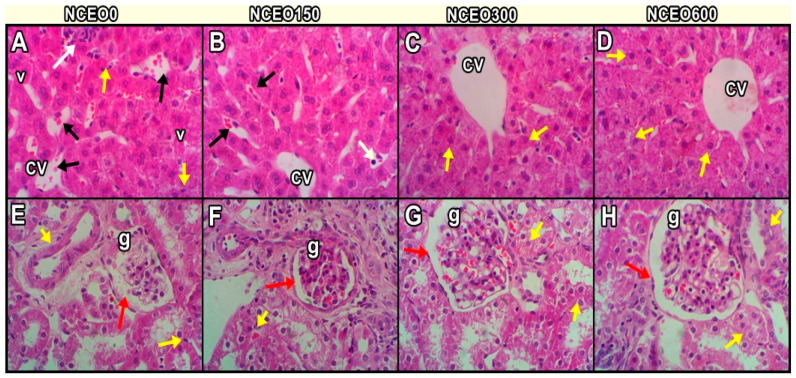
Representative photomicrographs of liver (**A**–**D**) and kidney (**E**–**H**) of growing rabbits. The hepatic structure of rabbits fed diets without supplement (**A**) showing dilated and congested central veins (CV), with a marked degree of hepatic necrosis (yellow arrow), associated mononuclear inflammatory cells (v) infiltration (white arrow), and degeneration of hepatocytes with large cytoplasmic vacuoles. The other treated groups; rabbit fed diets supplemented with 150 (**B**), 300 (**C**), and 600 (**D**) mg of NCEO/kg, showing normal organization of hepatic parenchyma consisting of radially arranged hepatic lobules around CV, normal hepatocytes with little vacuolated cytoplasm, and normal sinusoids (black arrows) and normal endothelial cells. Heat stress caused focal, coalescing mild fibrosis with moderate tubular damage (yellow arrow), intratubular aggregation of sloughed epithelium with moderately congested glomeruli (g), and atrophies (**E**) in the renal tissues of growing rabbits, while renal tissues of treated rabbit with NCEO at different levels 150 (**F**), showing multifocal mild cortical interstitial nephritis-separated degenerated tubules (yellow arrows) and observed atrophied glomerulus (g). Groups fed with 300 (**G**) and 600 (**H**) mg of NCEO/kg diet, showing mostly a normal histological appearance, normal tubules, and glomerulus (g) surrounded by visceral and parietal (red arrows) layers of Bowman’s capsule and separated by Bowman’s space. (Scale bar = 40×).

The liver tissues of the stressed growing rabbits (NCEO0; Figure 3A) exhibited dilated and congested central veins (CV), with a marked degree of hepatic necrosis (yellow arrow) and associated mononuclear inflammatory cell infiltration (white arrow). Moreover, the NCEO0 group displayed the degeneration of hepatocytes with large cytoplasmic vacuoles (v), sinusoidal dilatations, interstitial edema with Kupffer cells, and blood infiltration (black arrows). The growing rabbits fed with 150 mg/kg of NCEO exhibited a moderate improvement in their hepatic architectures (Figure 3B). A normal organization of hepatic parenchyma was seen, consisting of radially arranged hepatic lobules around CV, normal hepatocytes with little vacuolated cytoplasm, and normal sinusoids (black arrows) (Figure 3B). The hepatic sections of the growing rabbits in the groups (NCEO300; Figure 3C and NCEO600; Figure 3D) demonstrated normal hepato-portal structures, comprising the central vein and parenchymal cells, reducing the inflammatory cells induced by HS, as reported in NCEO0 (Figure 3A). Microscopically, HS can cause focal, coalescing mild fibrosis with moderate tubular damage (yellow arrow), an intra-tubular aggregation of sloughed epithelium with moderately congested glomeruli (g), and atrophies (Figure 3E) in the renal tissues of growing rabbits. The growing rabbits fed with 150 mg of NCEO/kg under HS conditions showed multifocal, mild cortical interstitial nephritis-separated degenerated tubules (yellow arrows) and observed atrophied glomerulus (g). The renal tissue sections of the growing rabbits in the NCEO300 (Figure 3G) and NCEO600 (Figure 3H) groups presented normal histological structures of their tubules, glomeruli, and interstitial tissue.

## 4. Discussion

The profitable production of rabbit meat is negatively affected by heat stress (HS), as rabbits’ growth requires a convenient climate for the expression of the good-quality traits embedded in the genetic components of rabbits [33,34]. Rabbits have few numbers of sweat glands, mainly under their feet and on their lips. These sweat glands are not effective at regulating exceeded body temperature, particularly in hot conditions, which makes them more sensitive to HS, which has adverse effects on their welfare, adaptation, feed efficiency, growth performance, redox status, inflammatory response, and health status [7,35,36], and also disrupts the fertility traits in rabbit does [7] and bucks [37]. The temperature–humidity index (THI) can be useful for assessing the risk of HS on rabbits induced by hot environments [38]. Even though the values of the THI in this study indicated that the growing rabbits were subjected to severe HS conditions [20], the NCEO-supplemented rabbits had a better capacity for heat tolerance, growth performance, redox balance, and health status. The fortification of the rabbit diets with NCEO resulted in a significant decrease in body temperature. This indicates that the bioactive components presented in NCEO, such as flavones and flavonoids, have thermoregulatory effects [6,17]. To achieve thermoregulation, there must be a balance between heat gain and heat loss in the animal body. Therefore, under severe HS conditions, rabbits begin to increase their panting rate (respiratory rate) to dissipate the heat load via the respiratory tract using evaporation, which explains the observed increment in breathing rate in the control group compared to the other treated groups. The thermoregulatory actions of some herbs have been confirmed in animals by Abd El-Hack et al. [39], due to their robust antioxidant properties.

The above results indicate that the better growth performance of the NCEO-supplemented rabbits was achieved by enhancing the defeasibility of nutrients, which provide available energy for growth and heat dissipation from the body. In this study, the NCEO300 and NCEO600 groups had a higher FBW, ADG, SGR, and PI and a lower FCR than the control and other treated groups, which is in general agreement with the findings of former studies [36,40]. They reported that the dietary supplementation of rabbit diets with nano-emulsified essential oils enhanced their growth performance and feed utilization.

The improvement in growth performance (higher LBW and ADG) in the NCEO-supplemented rabbits may be attributed to the presence of some bioactive constituents (saponins, tannins, and flavonoids), which promote the digestion of nutrients and their absorption, like other essential oils, by increasing the secretion of digestive enzymes and destroying infectious bacteria [39]. Another factor demonstrating the benefits of using NCEO was its stimulating effect on the animals’ digestive systems by increasing gastric acid secretion and representing a robust antimicrobial agent [41,42]. In the present study, the improvement in feed utilization (lower FCR) was triggered by larger increases in FBW rather than FI, which suggested that the NCEO-supplemented rabbits could more worthily utilize the diet supplemented with the NCEO [43]. In parallel, Omidi et al. [13] suggested that adding 50 mg of cardamom essential oils/kg diet can improve the ADG and FCR of broilers during the grower period (11–28 days).

Hematological attributes are considered to be good indicators for rabbits’ general health status, immune capacity, infectious diseases, and/or other environmental issues such as HS. High temperatures can disrupt the hematological variables, particularly the leucocyte and erythrocyte counts, making animals more sensitive to infection or disease. In this sense, Hashem et al. [44] clarified that HS can lessen the leucocyte count levels, resulting in immune dysfunction through the altitude of WBC counts in the blood of rabbits. The addition of NCEO led to a significant increase in erythrocyte count, including PLT and RBC counts, as well as the concentration of HGB. Moreover, the dietary NCEO supplementation significantly decreased the WBC count, being in the normal range that reflected an improved health status of the growing rabbits exposed to a high ambient temperature.

In the current study, the relative weight of the liver was significantly higher in both the NECO300 and NECO600 groups than that in the control and NECO150 groups, reflecting the improved health status of the growing rabbits, as the liver has a crucial role in the synthesis of the enzymes related to heat tolerance, as well as blood protein synthesis [7,10,33]. In this context, despite the rapid metabolization of essential oils, they can damage the liver and thus increase the blood content of their enzymes [45]. Still, the lower serum contents from the liver enzymes (AST and ALT) and decreased urea and creatinine concentrations in our study showed that the administration of NCEO oil was safe and helpful for expressing liver and renal functions. In correspondence with this study, Traesel et al. [46] reported that the extended use of high concentrations of essential oils could not cause renal and/or nephritis failure.

It is well-known that blood biochemical parameters can reflect the physiological responses of rabbits to their external and internal environments. Even though the TP of serum and its fraction usually decrease during HS [20], in this study, there was a significant increase in the blood TP and albumin of the NCEO-supplemented rabbits, reflecting the good nutritional status of the treated groups. Concerning the blood glucose concentration, it increased markedly with a high ambient temperature [47], and this may have been due to the depression of feed intake and subsequent reduction in metabolic rate [37,48]. In addition, Kiwull-Schöne et al. [49] demonstrated that increased blood glucose levels were directly correlated with HS inducing the release of glucocorticoids to the blood. Herein, the decreasing blood glucose concentrations shown in the NCEO300 group could be explained by the greater activity of blood insulin [50]. Our data are in harmony with Khalid et al. [50], who reported that adding natural phytochemicals to heat-stressed growing rabbits’ diets significantly decreased the blood serum glucose.

Regarding the lipid profile, previous investigations have revealed favorable effects of CEO on cholesterol metabolism [13]. There is a lack of research investigating CEO’s impacts on rabbits’ lipid profiles. Still, in parallel, the dietary supplementation of broilers’ diets with spice additives has been observed to have a hypocholesterolemic effect, as there were significant decreases in the concentrations of triglycerides, cholesterol, and low-density lipoproteins. Meanwhile, there was a significant increase in the concentration of high-density lipoprotein. Our results revealed that the dietary addition of 300 or 600 mg of NCEO/kg diet showed a potent biological effect on decreasing the concentrations of serum triglycerides and cholesterol compared to those in the untreated group. The present results may be explained by the fact that spice additives can regulate the production of cholesterol and exert a hypocholesterolemic effect throughout the inhibition of the 3-hydroxy-3-methyl-glutaryl-CoA reductase [51].

It is worth noting that there was overproduction from reactive oxygen species (ROS) under HS conditions, which induced oxidative stress and impaired the function and structure of important molecules such as proteins and nucleic acids [52]. The production of ROS is balanced by the antioxidant defense system, comprising enzymes such as SOD and GSH. Several former scientific researchers have observed that a high ambient temperature ordinarily increases oxidative stress by increasing lipid oxidation and/or proteins (protein carbonyl) and disrupting antioxidant enzyme production [53,54]. In the present study, the dietary administration of NCEO improved the indices of anti-oxidative stress; the NCEO300 group showed higher concentrations of SOD and GSH, and lower concentrations of MDA and PC, than those in the control. Moreover, the TAC increased by 67.65 and 102.67% in the NECO300 and NECO600 groups compared to that in the control. The present study agreed with numerous previous studies that deduced that essential oils could alleviate the negative effects of HS and achieve oxidative stability by inhibiting the diffusion of oxidation reactions and effectually deferring the oxidation of lipids and/or proteins and other nutrients [7,55].

The upper levels of pro-inflammatory cytokines, such as IFN-γ and IL-4, are induced in the blood serum along with an elevated ambient temperature, increasing the intestinal permeability to pathogens [39]. Our study exhibited noteworthy decreases in the concentrations of pro-inflammatory cytokines (IFN-γ and IL-4) in the NCEO-supplemented rabbits compared to the control, indicating the potent anti-inflammatory activities of NCEO. On the other hand, the dietary treatment resulted in a significant increase in the concentration of NO, which is a small molecule that plays an indispensable role under physiological concentrations in defense against pathogens and thermos-tolerance during heat stress, as it has a decisive role in the vasodilation of the blood vessels of the skin [48,56,57]. Moreover, mounting evidence suggests that NO plays other roles in neurotransmission and immune regulation [7,57]. The anti-inflammatory effect of NCEO is attributed to the presence of 1,8-cineole and α-terpinyl acetate [14]. It was interesting to obtain an increased concentration of NO in the blood serum of the NCEO-supplemented rabbits, which is in line with the increased concentrations of the antioxidant indices (GSH, SOD, and TAC), indicating that the NO concentration was in the normal physiological range, as it is classified as a free radical as well.

Concerning general health status, HS is involved in shrinking newly weaned rabbits’ immune capacity, due to its ability to promote the synthesis of immunological variables such as IgM, IgA, and IgG [36]. Further, the impairment of the immune response could make growing rabbits more sensitive to pathogens [58,59]. This immune imbalance caused by HS certainly delays the growth of growing rabbits [10]. In the current study, the NCEO supplementation could promote the synthesis of immunological variables, including IgG and IgM, effectively overcoming the detrimental impacts of HS on newly weaned rabbits during summer. The affirmative effects of NCEO could be attributed to its antibacterial, antioxidant, and anti-inflammatory properties [13]. These phytogenic additives can minimize bacteria colonization and hinder the growth of pathogenic and/or non-pathogenic strains of bacteria in the gut of rabbits [42]; also, they can bring the ecosystems of microbes in the rabbit gut to equilibrium, leading to a better feed efficiency and metabolism [52]. Studying the pathological alteration of the main organs in the body such as liver and kidney behind the blood chemistry may provide more insights into the potential of using NCEO for improving the functionality of renal/hepato cells in growing rabbits. As clarified in this research, NECO successfully re-established and recovered the near-normal renal–hepatic morphology induced by HS. In our previous work, we observed that some natural pigments (prodigiosin) enhanced the structure of the hepatic morphology in stressed growing rabbits [58] by reducing the TNF-α in hepatic tissues. Recently, the renal-protective effect of cardamom essential oil was evidenced by [15]. The potential of CNEO to sustain the renal histological profile could explain its protective action against HS. Transcriptomics and proteomics explorations should be performed during HS to provide insights into suitable strategies for mitigating HS in animals.

## 5. Conclusions

Based on the results mentioned above, the dietary supplementation of heat-stressed growing rabbit’s diets with NECO could conquer the adverse effects of natural heat stress on growth performance, feed utilization, antioxidants criteria, immunity status, and inflammatory response, thus enhancing the general health statuses of the growing rabbits. To support their commercial production, upcoming investigations are needed to identify the major biological features in nano-emulsion essential oils.

## Figures and Tables

**Table 1:** Formulation and chemical analysis of the basal diet fed to the growing rabbits.

Ingredients	%
Alfalfa hay	34
Wheat bran	21
Soybean meal	15
Barley grains	17
Yellow corn	8
Cane molasses	3
Dicalcium phosphate	1
Sodium chloride	0.5
DL-methionine	0.1
Mineral and vitamin mixture *	0.4
Chemical analysis	
Organic matter	91.27
Crude protein (n × 6.25)	18.4
Crude fiber	14.32
Ether extract	2.98
Digestible energy (kcal/kg)	2507
Metabolizable energy(kcal/kg)	2321

* Premix containing the following vitamins and minerals (per kilogram of diet): vitamin A, 8000 IU; vitamin D3, 600 IU; vitamin E, 34 mg; vitamin K3, 1.32 mg; vitamin B1, 1.32 mg; vitamin B2, 4.0 mg; vitamin B6, 1.32 mg; vitamin B12, 0.01 mg; pantothenic acid, 13.32 mg; biotin, 0.13 mg; folic acid, 3.32 mg; choline chloride, 800 mg; manganese sulfate, 32 mg; zinc oxide, 60 mg; iron carbonate, 120 mg; copper oxide, 16 mg; potassium iodide, 2 mg; sodium selenite, 0.4 mg; and cobalt oxide, 0.4 mg.

**Table 2:** The mean values of temperature–humidity index, relative humidity, and ambient temperature during the trial duration.

Parameters	July	August	Overall
AT (°C)	31.19 ± 0.21	31.12 ± 0.17	31.16 ± 0.07
RH (%)	72.31 ± 0.63	71.96 ± 0.44	72.13 ± 0.21
THI	29.74 ± 0.09	29.66 ± 0.11	29.70 ± 0.10

RH: relative humidity; AT: ambient temperature; and THI: temperature–humidity index.

**Table 3:** Growth performance and feed utilization of growing V-line rabbits exposed to high ambient temperature as affected by different levels of NCEO.

Variables	NCEO0	NCEO150	NCEO300	NCEO600	SEM	*p*-Value
Live body weight (g)						
5 wks of age	655.60	652.53	649.33	658.20	4.881	0.6069
9 wks of age	1399.00 ^c^	1450.53 ^b^	1469.27 ^b^	1514.13 ^a^	8.764	<0.0001
13 wks of age	2056.33 ^c^	2120.33 ^b^	2130.20 ^ab^	2173.87 ^a^	16.176	<0.0001
Average daily gain (g)						
5–9 wks of age	26.55 ^d^	28.50 ^c^	29.18 ^b^	30.07 ^a^	0.224	<0.0001
9–13 wks of age	23.48	23.92	23.60	23.56	0.498	0.9281
5–13 wks of age	25.01 ^c^	26.21 ^b^	26.44 ^ab^	27.07 ^a^	0.274	<0.0001
Feed intake (g/day)						
5–9 wks of age	73.60	71.63	73.13	69.53	2.628	0.4877
9–13 wks of age	117.60	116.53	118.80	117.60	1.992	0.8848
5–13 wks of age	96.87	94.27	93.01	89.2	3.123	0.7077
Feed conversion ratio (g feed/g gain)						
5–9 wks of age	2.80 ^a^	2.51 ^b^	2.50 ^b^	2.29 ^c^	0.056	<0.0001
9–13 wks of age	5.02	4.87	5.04	5.03	0.126	0.9182
5–13 wks of age	3.87 ^a^	3.60 ^ab^	3.52 ^ab^	3.30 ^b^	0.117	0.0495

^a,b,c,d^ Means within a row without a common superscript letter differ at *p* < 0.05. Rabbit-fed diets have different levels of nanoemulsion of cardamom essential oil at 0 (NCEO0), 150 (NCEO150), 300 (NCEO300), and 600 (NCEO600) mg/kg diet, respectively.

**Table 4:** Carcass traits of growing V-line rabbits exposed to high ambient temperature as affected by different levels of NCEO.

Items	NCEO0	NCEO150	NCEO300	NCEO600	SEM	*p*-Value
Pre-slaughter weight (g)	2088.60	2095.40	2114.40	2087.00	13.15	0.4565
Carcass Traits (as % of pre-slaughter BW)						
Dressing	55.23 ^b^	58.32 ^ab^	60.66 ^a^	58.57 ^ab^	1.134	0.0290
Head	4.67	4.87	5.24	5.09	0.19	0.2089
Liver	2.85 ^b^	3.23 ^ab^	3.59 ^a^	3.55 ^a^	0.169	0.0239
Heart	0.29	0.32	0.34	0.32	0.032	0.8896
Kidney	0.87	0.94	0.92	0.92	0.054	0.8620
Edible Giblets	4.01 ^b^	4.49 ^ab^	4.85 ^a^	4.78 ^a^	0.22	0.0104
Spleen	0.07	0.10	0.07	0.10	0.017	0.1938
Lung	0.69	0.69	0.75	0.70	0.047	0.7879
Cecum Length (Cm)	12.28	11.84	12.60	12.63	0.729	0.8096

^a,b^ Means within a row without a common superscript letter differ at *p* < 0.05. Rabbit-fed diets having different levels of nanoemulsion of cardamom essential oil at 0 (NCEO0), 150 (NCEO150), 300 (NCEO300), and 600 (NCEO600) mg/kg diet, respectively. Edible giblets consist of the heart, kidney, and liver.

**Table 5:** Blood hematology of growing V-line rabbits exposed to high ambient temperature as affected by different levels of NCEO.

Variables	NCEO0	NCEO150	NCEO300	NCEO600	SEM	*p*-Value
HGB (g/dL)	8.67 ^b^	9.07 ^b^	10.54 ^a^	10.63 ^a^	0.423	0.0221
RBCs (10^6^/μL)	5.84 ^b^	6.39 ^ab^	6.28 ^ab^	7.31 ^a^	0.337	0.0459
PLT (10^3^/mm^3^)	275.85 ^b^	344.61 ^a^	345.92 ^a^	340.01 ^ab^	19.679	0.0369
HCT (%)	32.59 ^b^	32.73 ^b^	32.73 ^b^	43.90 ^a^	1.348	0.0007
MCV (fl)	67.04	68.93	67.48	70.79	3.233	0.8427
MCH (pg)	20.11	20.77	24.15	20.48	4.557	0.9148
MCHC (g/dL)	31.76	31.13	30.10	31.03	7.927	0.9990
WBCs (10^3^/mm^3^)	7.34 ^a^	6.14 ^ab^	5.56 ^b^	4.81 ^b^	0.439	0.0200
Leucocytes fractions (%)						
LYM	33.29	32.68	32.72	31.33	1.263	0.7357
MON	8.81	9.92	9.11	8.02	0.710	0.3651
NEUT	53.66	53.15	54.27	53.87	1.416	0.9527
ESIN	2.81	2.59	2.44	2.69	0.530	0.9666

Rabbit-fed diets having different levels of nanoemulsion of cardamom essential oil at 0 (NCEO0), 150 (NCEO150), 300 (NCEO300), and 600 (NCEO600) mg/kg diet, respectively. HGB, hemoglobin; RBCs, red blood corpuscles; PLT, platelet count; HCT, hematocrit; MCV, mean corpuscular volume; MCH, mean corpuscular hemoglobin; MCHC, mean corpuscular hemoglobin concentration; WBCs, white blood cells count; LYM, lymphocytes; MONO, monocytes; NEUT, Neutrophils; and ESIN, eosinophils. ^a,b^ Means within a row without a common superscript letter differ at *p* < 0.05.

**Table 6:** Blood profile of growing V-line rabbits exposed to high ambient temperature as affected by different levels of NCEO.

Variables	NCEO0	NCEO150	NCEO300	NCEO600	SEM	*p*-Value
TP (g/dL)	5.803 ^b^	6.057 ^b^	6.757 ^a^	7.043 ^a^	0.126	0.0004
Alb (g/dL)	3.467 ^b^	3.160 ^c^	3.777 ^a^	3.627 ^ab^	0.087	0.0058
Glob (g/dL)	2.337 ^c^	2.897 ^b^	2.980 ^b^	3.417 ^a^	0.052	<0.0001
Alb/Glob ratio	1.485 ^a^	1.091 ^c^	1.267 ^b^	1.061 ^c^	0.025	<0.0001
AST (IU/L)	44.313 ^a^	40.407 ^a^	34.230 ^b^	35.790 ^b^	1.263	0.0019
ALT (IU/L)	72.550 ^a^	60.867 ^b^	60.637 ^b^	54.870 ^b^	1.907	0.0012
TB (mg/dL)	0.823	0.813	0.757	0.793	0.051	0.8077
GGT (mg/dL)	37.910	37.520	38.240	37.960	1.244	0.9810
Uric acid (mg/dL)	1.810 ^a^	1.350 ^b^	1.103 ^b^	1.320 ^b^	0.083	0.0021
Creatinine (mg/dL)	1.160 ^a^	0.813 ^b^	0.750 ^bc^	0.650 ^c^	0.037	<0.0001
Glucose (mg/dL)	109.63 ^a^	106.96 ^a^	94.62 ^b^	91.33 ^b^	1.338	0.0001
TG (mg/dL)	86.240 ^a^	79.050 ^b^	71.593 ^c^	63.117 ^c^	1.634	0.0005
TC (mg/dL)	100.883 ^a^	80.717 ^b^	84.787 ^b^	82.670 ^b^	1.749	0.0001
LDL (mg/dL)	48.279 ^a^	25.470 ^b^	31.541 ^b^	24.710 ^b^	4.496	0.0197
HDL (mg/dL)	30.690 ^b^	39.437 ^a^	38.927 ^a^	45.337 ^a^	2.416	0.0255
VLDL (mg/dL)	17.248 ^a^	15.810 ^b^	14.319 ^c^	12.623 ^d^	0.326	0.0005

Rabbit-fed diets having different levels of nanoemulsion of cardamom essential oil at 0 (NCEO0), 150 (NCEO150), 300 (NCEO300), and 600 (NCEO600) mg/kg diet, respectively. TP, total protein; Alb, albumin; Glob, globulin; AST, aspartate aminotransferase; ALT, alanine aminotransferase; TB, total bilirubin; GGT, glutamyl transferase; TG, triglycerides;TC, total cholesterol; LDL, low-density lipoprotein; HDL, high-density lipoprotein; and VLDL, very low density lipoprotein ^a,b,c,d^ Means within a row without a common superscript letter differ at *p* < 0.05.

**Table 7:** Redox status of growing V-line rabbits exposed to high ambient temperature as affected by different levels of NCEO.

Variables	NCEO0	NCEO150	NCEO300	NCEO600	SEM	*p*-Value
TAC (U/mL)	1.008 ^c^	1.437 ^c^	1.890 ^b^	2.243 ^a^	0.074	<0.0001
SOD (ng/mL)	4.551 ^c^	4.660 ^c^	5.370 ^a^	5.061 ^b^	0.080	<0.0001
GSH (ng/mL)	0.188 ^b^	0.294 ^a^	0.318 ^a^	0.290 ^a^	0.013	0.0005
MDA (nmol/mL)	0.654 ^a^	0.634 ^a^	0.492 ^b^	0.503 ^b^	0.024	0.0027
PC (nmol/mL)	4.543 ^a^	4.096 ^b^	1.825 ^d^	3.117 ^c^	0.107	<0.0001

Rabbit-fed diets have different levels of nanoemulsion of cardamom essential oil at 0 (NCEO0), 150 (NCEO150), 300 (NCEO300), and 600 (NCEO600) mg/kg diet, respectively. TAC, total antioxidant capacity; SOD, superoxide dismutase; GSH glutathione; MDA, malondialdehyde; and PC, protein carbonyl; ^a,b,c,d^ Means within a row without a common superscript letter differ at *p* < 0.05.

**Table 8:** Inflammatory responses and immunity status of growing V-line rabbits exposed to high ambient temperature as affected by different levels of NCEO.

Variables	NCEO0	NCEO150	NCEO300	NCEO600	SEM	*p*-Value
Inflammatory responses						
IFN-γ (pg/mL)	83.75 ^a^	81.46 ^a^	58.06 ^b^	59.33 ^b^	3.213	0.0164
IL-4 (pg/mL)	83.22 ^a^	70.54 ^b^	67.80 ^bc^	60.80 ^c^	2.814	0.0032
NO (Umol/L)	35.66 ^c^	45.28 ^b^	53.63 ^a^	49.47 ^ab^	2.482	0.0012
Immunity status						
IgG (mg/mL)	467.12 ^b^	541.10 ^a^	549.80 ^a^	547.51 ^a^	12.190	0.0041
IgM (mg/mL)	101.47 ^c^	118.41 ^b^	130.55 ^b^	174.92 ^a^	4.446	<0.0001

Rabbit-fed diets have different levels of nanoemulsion of cardamom essential oil at 0 (NCEO0), 150 (NCEO150), 300 (NCEO300), and 600 (NCEO600) mg/kg diet, respectively. INF-γ, interferon γ; IL4: interleukin-4; NO: nitric oxide; IgG, immunoglobulin G; and IgM, immunoglobulin M; ^a,b,c^ Means within a row without a common superscript letter differ at *p* < 0.05.

## Data Availability

The data presented in this study are available on request from the corresponding author.
